# Rumen Fermentation Parameters Prediction Model for Dairy Cows Using a Stacking Ensemble Learning Method

**DOI:** 10.3390/ani13040678

**Published:** 2023-02-15

**Authors:** Yuxuan Wang, Jianzhao Zhou, Xinjie Wang, Qingyuan Yu, Yukun Sun, Yang Li, Yonggen Zhang, Weizheng Shen, Xiaoli Wei

**Affiliations:** 1 College of Electric and Information, Northeast Agricultural University, Harbin 150030, China; 2 College of Animal Sciences and Technology, Northeast Agricultural University, Harbin 150030, China

**Keywords:** methane, volatile fatty acid, rumen metabolism, total mixed ration, dairy cattle

## Abstract

**Simple Summary:**

Methane and volatile fatty acids are important products of rumen fermentation in dairy cows. Quantitative research on them is of great significance for environmental protection and animal production. The aim of this study was to develop a prediction model using the stacking ensemble learning method and predict the production of rumen fermentation products based on the nutrient level of the diet. The results show that the stacking model has good performance in terms of prediction accuracy. The model proposed in this study can be used as a guideline to optimize diet compositions and improve feeding efficiency.

**Abstract:**

Volatile fatty acids (VFAs) and methane are the main products of rumen fermentation. Quantitative studies of rumen fermentation parameters can be performed using in vitro techniques and machine learning methods. The currently proposed models suffer from poor generalization ability due to the small number of samples. In this study, a prediction model for rumen fermentation parameters (methane, acetic acid (AA), and propionic acid (PA)) of dairy cows is established using the stacking ensemble learning method and in vitro techniques. Four factors related to the nutrient level of total mixed rations (TMRs) are selected as inputs to the model: neutral detergent fiber (NDF), acid detergent fiber (ADF), crude protein (CP), and dry matter (DM). The comparison of the prediction results of the stacking model and base learners shows that the stacking ensemble learning method has better prediction results for rumen methane (coefficient of determination ($R^{2}$) = 0.928, root mean square error (RMSE) = 0.968 mL/g), AA ($R^{2}$ = 0.888, RMSE = 1.975 mmol/L) and PA ($R^{2}$ = 0.924, RMSE = 0.74 mmol/L). And the stacking model simulates the variation of methane and VFAs in relation to the dietary fiber content. To demonstrate the robustness of the model in the case of small samples, an independent validation experiment was conducted. The stacking model successfully simulated the transition of rumen fermentation type and the change of methane content under different concentrate-to-forage (C:F) ratios of TMR. These results suggest that the rumen fermentation parameter prediction model can be used as a decision-making basis for the optimization of dairy cow diet compositions, rapid screening of methane emission reduction, feed beneficial to dairy cow health, and improvement of feed utilization.

## 1. Introduction

Methane is a greenhouse gas with a long half-life. Methane emissions from ruminants are an important source of anthropogenic greenhouse gases. Volatile fatty acids (VFAs) affect the health of dairy cows. The production of VFAs in the rumen can cause changes in milk production and composition [1]. Excessive VFAs can cause the rumen buffer to fail, affect the activity of rumen microorganisms, make dairy cows have a poor appetite, and even lead to subacute ruminal acidosis [2]. Methane and VFAs are important products of rumen fermentation, and their production can reflect important information on the nutritional utilization of ruminants [3]. Methane production indicates energy loss in ruminants, while VFAs are an energy source for ruminants, accounting for about 40–70% of digestible energy intake [4]. The quantitative methods for rumen fermentation products from ruminants are of great importance, whether from the perspective of environmental protection or animal production.

Methane and VFAs can be measured directly by an in vivo technique, but the measurements are time-consuming, laborious, expensive, and unsuitable for large-scale feed evaluation [5]. The in vitro technique can well simulate rumen fermentation and predict the digestion of feed in ruminants, and the test results are closely related to the in vivo technique [6]. Shi et al. [7] used in vitro technology to simulate rumen fermentation and measure the fermentation parameters of corn straw. Weiby et al. [8] found that changes in feed quality parameters were associated with methane production determined using the in vitro method. In our previous study, the relationship between rumen fermentation parameters using total mixed rations (TMRs) and dairy cow production performance was found using the in vitro method. However, in vitro technology has the drawbacks of heavy dependence on fistula animals and complicated operation processes. Machine learning methods are also used for the prediction of rumen fermentation parameters [9,10]. Hempel et al. [11] compared and evaluated the accuracy and robustness of several supervised machine learning methods in predicting methane emissions from a dairy cattle barn. Li et al. [10] used artificial neural networks to predict multiple rumen fermentation parameters. However, Hristov et al. [12] pointed out that the robustness of the existing prediction models for methane emissions is poor due to the small data sets. The stacking ensemble learning model fits the need for high robustness and generalization ability in the case of small samples. Previous studies tended to compare different models and choose the one with better performance, while the stacking ensemble learning method can weigh the contribution of each prediction model and provide a new integrated model with improved performance and high robustness.

Many scholars have discussed the correlation between diet composition and rumen fermentation [13,14,15]. Based on the previous research, this study uses data collected by the in vitro method and selects neutral detergent fiber (NDF), acid detergent fiber (ADF), crude protein (CP), and dry matter (DM) as model input factors. In this study, it was hypothesized that the rumen fermentation parameters prediction model can be constructed using the stacking ensemble learning method. The objectives of this study were to build a stacking model to predict the production of rumen fermentation products (methane, acetic acid (AA), and propionic acid (PA)), compare the accuracy and precision of the stacking model and the base learners under multiple evaluation methods, and demonstrate the generalization ability of the model under the condition of small samples through an independent validation experiment.

## 2. Materials and Methods

### 2.1. Data Collection

To accurately predict the rumen fermentation parameters of dairy cows, this study obtained the sample data required for the prediction model through actual feeding and digestion experiments. Different TMR (taken from different farms in Heilongjiang, China, in 2018) were collected for the in vitro fermentation experiment. Multi-point collection was carried out after feeding by the feeding truck, and (500 ± 50 g) TMR samples were obtained by the quadrat method. The TMR were dried at 65 °C for 48 h, ground into 40 meshes, weighed 0.2 g, and loaded into a fiber bag as a fermentation substrate. The nutrient levels (NDF, ADF, CP, and DM) of TMR were measured according to the literature [16]. Rumen fluid was collected from two Holstein cows in good condition and weighing (650 ± 20 kg) with permanent rumen fistulae. Take 30 mL of the rumen fluid that was buffered into the prewarmed incubation flasks. Each incubation flask was incubated at 39 °C in water. The methane, AA, and PA contents of all samples were measured after 24 h of in vitro fermentation. In the gas collection bag, the methane content was measured. The levels of VFAs were measured by gas chromatography. All analyses were conducted in quintuplicate. More details about the experiment with the methane and VFAs collection can be found in the literature [16].

**Figure 1:**
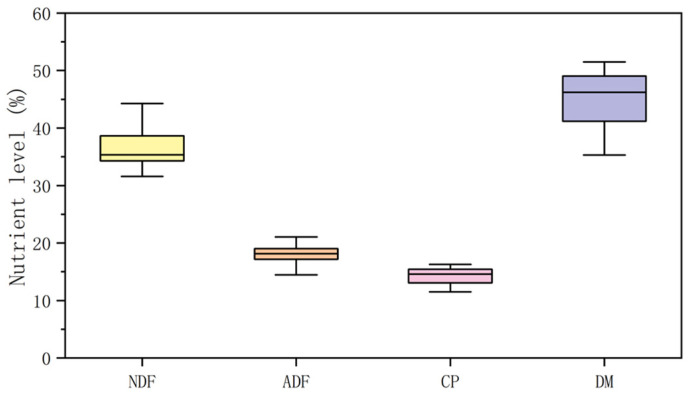
Boxplot of the nutrient levels (neutral detergent fiber (NDF), acid detergent fiber (ADF), crude protein (CP), and dry matter (DM)) in the dataset.

The experiment was completed at the A-cheng Experimental Base (Harbin city, China) of Northeast Agricultural University in 2018. A total of 120 groups of data were obtained, and the nutrient levels are shown in Figure 1. The whole data set was randomly split into a training data set and a test data set. 85% of the data were used for training the model, and the remaining 15% of the data were used for model testing.

### 2.2. Data for Validation Experiments

To prove the robustness and strong generalization ability of the prediction model proposed in this paper, an independent validation experiment was conducted in Changchun, China (2021). Different diet compositions and rumen fluid were used in this experiment. Rumen fluid was collected from three dairy cows weighing (500 ± 50 kg) before the morning feeding. The in vitro incubation procedure was the same as the previous study. The methane production was determined by a real-time in vitro fermentation system (produced by the Jilin Academy of Agricultural Sciences, code Qtfxy-6) [17]. Nitrogen was passed into the incubation flask from the bottom. Methane was carried by nitrogen into an AGM10 sensor (Sensors Europe GmbH, Erkrath, FRG), and then the methane content was measured. For the analysis of AA and PA, 1 mL of 25% meta-phosphoric acid was added to 5 mL of culture liquor and stored at −20 °C. AA and PA contents were measured by gas chromatography. All analyses were conducted in triplicate. In this validation experiment, TMR were prepared using the compound feed from the Ruminant Nutrition Laboratory at Northeast Agriculture University. Studies have proven that the concentrate-to-forage (C:F) ratio of the diet is the main factor that changes the type of rumen fermentation and affects rumen methane production [8,18]. To validate the model’s simulation of this problem, two TMRs were selected for the validation experiment, namely T_1_ (C:F ratio of 40:60) and T_2_ (C:F ratio of 60:40). Both T_1_ and T_2_ met the animals’ requirements for nutrition based on the CPM-Dairy model (Cornell-Penn-Miner Dairy, version 3.08.01) [19]. The ingredients and chemical composition are shown in Table 1.

### 2.3. Modeling

Ensemble learning is a popular machine learning strategy. There are three core ideas in ensemble learning: bagging, boosting, and stacking. Ensemble learning combines multiple models, and the error of a single base learner is likely to be compensated by other base learners. Therefore, the overall prediction performance will be better than that of a single base learner [20]. Stacking is a prominent method for forming linear combinations of different predictors to provide improved prediction accuracy. The idea is to use cross-validation data and least squares under non-negativity constraints to determine the coefficients in the combination. Essentially, a stacked model works by running the output of multiple models through a “meta-learner” (usually a linear regressor/classifier). The meta-learner attempts to minimize the weaknesses and maximize the strengths of every individual model. The final result is usually a very robust model. In our study, a stacking model based on gradient boosting regression tree (GBRT), Gaussian process regression (GPR), and bagging regression (BR) has been developed to predict the rumen fermentation parameter values of dairy cows.

#### 2.3.1. Base-Level Learning Component

GBRT is one of the boosting-based ensemble learning methods proposed by Friedman [21]. GBRT mainly consists of two parts: the gradient boosting (GB) algorithm and the regression tree (RT) algorithm. GB: boosting makes common decisions by iterating over multiple trees. The core idea is that each tree is the conclusion and residual of all trees before learning. RT: the GBRT algorithm combines many weak learners to come up with one strong learner. The weak learners here are generated by the classification and regression trees (CART) algorithm, which was first introduced by Breiman et al. [22].

The generic GBRT method is: first input training set samples $\left\{\left(x_{1},y_{2}),\;(x_{1},y_{2}),\;\cdots ,\;(x_{i},y_{i}\right)\right\},\;\left(i=1,2,3,\cdots ,n\right)$ and set the loss function L(y,F(x)), number of iterations M.

Initialize weak learners:

(1) $$F_{0}\left(x\right)=\arg\min\overset{n}{\underset{i=1}{\sum }}L\left(y_{i},c\right)$$

For m=1 to M: compute so-called pseudo-residuals:

(2) $$r_{mi}=-{\left[\frac{\partial L\left(y_{i},F\left(x_{i}\right)\right)}{\partial F\left(x_{i}\right)}\right]}_{F\left(x\right)=F_{m-1}\left(x\right)}\mathrm{for}\;i=1,\ldots ,n.$$

Using $(x_{i},r_{mi})\;\left(i=1,2,3,\cdots ,n\right)$, a CART regression tree can be fitted, and the mth regression tree is obtained, with the corresponding leaf node region $R_{mj},\;j=1.2,\cdots ,J$. Where J is the number of leaf nodes on the regression tree.

For each sample in each leaf node, the output value $C_{mj}$ that minimizes the loss function and fits the leaf node best is found as follows.

(3) $$C_{mj}=\arg\min\underset{x_{i}\in R_{mj}}{\sum }L\left(y_{i}\cdot F_{m-1}\left(x_{i}\right)+c\right)$$

Update the strong learner:

(4) $$F_{m}\left(x\right)=F_{m-1}\left(x\right)+\overset{J}{\underset{j=1}{\sum }}C_{mj}I\left(x\in R_{mj}\right)$$

This leads to the final strong learner expression:

(5) $$F\left(x\right)=F_{0}\left(x\right)+\overset{M}{\underset{m=1}{\sum }}\overset{J}{\underset{j=1}{\sum }}C_{mj}I\left(x\in R_{mj}\right)$$

GPR is a machine learning regression method developed in recent years with a rigorous statistical theoretical foundation that is well adapted to handle complex problems with high dimensionality, small samples, and nonlinearity and has a high generalization capability. Compared with other machine learning techniques, the advantages of GPR are its seamless integration of various tasks, including parameter estimation, model training, and uncertainty estimation [23]. The method has the characteristics of easy implementation, adaptive acquisition of hyperparameters, and probabilistic significance of the output. GPR uses a Gaussian process (GP) prior to the regression analysis of the data and is a non-parametric model.

A GP is defined by its mean and covariance function (kernel function). In GPR, the data can be divided into training data X with the corresponding known output y and testing data $X^{*}$ with the corresponding unknown output $y^{*}$. Their prior mean function m(x) and covariance function $K\left(x,x^{\prime }\right)$ are expressed as the joint distribution:

(6) $$\left[\begin{array}{c} y\\ y^{*} \end{array}\right]\sim N\left(\left[\begin{array}{c} m\left(X\right)\\ m\left(X^{*}\right) \end{array}\right],\left[\begin{array}{cc} K\left(X,X\right) & K\left(X,X^{*}\right)\\ K\left(X^{*},X\right) & K\left(X^{*},X^{*}\right) \end{array}\right]\right)$$

The form of the mean function and covariance kernel function in the GP prior is chosen and adjusted during the model selection process. The mean function is typically constant, either zero or the mean of the training dataset. There are many options for the covariance kernel function: it can have many forms as long as it follows the properties of a kernel (i.e., is semi-positive, definite, and symmetric). In this study, a combination of a constant kernel and a radial basis function (RBF) kernel is used as the kernel function.

(7) $$K\left(x,x^{\prime }\right)=\sigma_{f}^{2}exp\left(-\frac{1}{2l^{2}}{\left\|x-x^{\prime }\right\|}^{2}\right)$$

This kernel has two hyperparameters: signal variance $\sigma^{2}$, and length scale l.

Bagging [24] is a well-known parallel ensemble learning method. The bagging algorithm can improve the accuracy of the model, reduce the variance of the results, and avoid overfitting. The bagging ensemble learning method takes several weak models, aggregating the predictions to select the best prediction. The weak models specialize in distinct sections of the feature space, which enables the bagging algorithm to use the predictions of each model to make the final prediction.

Bagging is composed of two parts: bootstrapping and aggregation. Bootstrapping is a sampling method in which a sample is chosen from a set using the replacement method. The learning algorithm is then run on the selected samples. The bootstrapping technique uses sampling with replacements to make the selection procedure completely random. When a sample is selected without replacement, the subsequent selections of variables are always dependent on the previous selections, making the criteria non-random. Model predictions undergo aggregation to combine them for the final prediction, which considers all the possible outcomes. The aggregation can be done based on the total number of outcomes or the probability of predictions derived from the bootstrapping of every model in the procedure.

The bagging algorithm uses the model averaging strategy. The reason why model averaging works is that different models usually do not produce the same error on the test set. Model averaging is a powerful and reliable method for reducing generalization error.

#### 2.3.2. Meta-Level Combining Component

The random sample consensus (RANSAC) algorithm takes the linear regression algorithm to the next level by excluding the outliers in the training dataset. RANSAC regression is a non-deterministic algorithm that tries to separate the training data into inliers and outliers. Outliers can come from extreme values of the noise, erroneous measurements, or incorrect hypotheses about the interpretation of data. Outliers have no value for the estimation of the model, and the final result of the RANSAC algorithm is obtained based on the set of inliers determined by the algorithm.

RANSAC regression is a robustness regression. One of the advantages of RANSAC is its ability to provide robust estimates of the model parameters, i.e., it can estimate the parameters with a high degree of accuracy even when a significant number of outliers are present in the data set. The RANSAC algorithm was first proposed by Fischler and Bolles [25].

#### 2.3.3. Stacking Model

**Figure 2:**
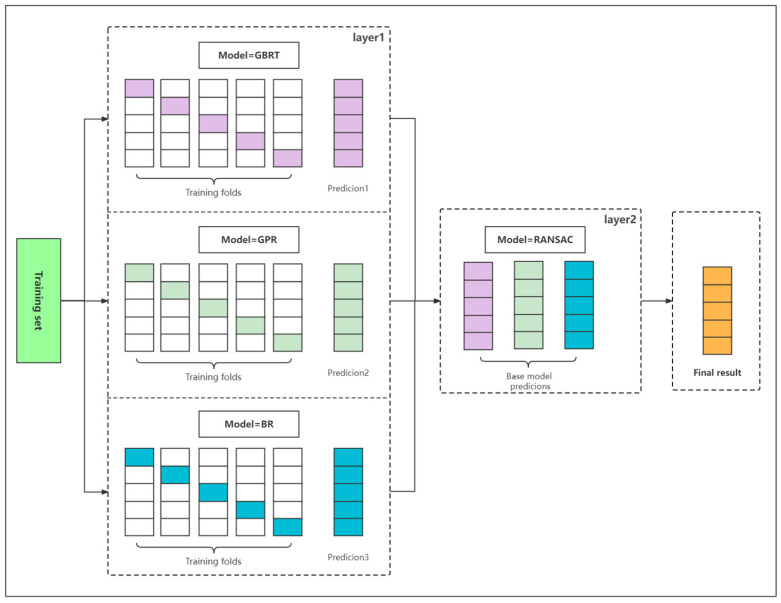
The stacking ensemble learning framework of this study. The first layer (the base-level learning component) consists of a gradient boosting regression tree (GBRT), a gaussian process regression (GPR), and a bagging regression (BR). The second layer (meta-level combining component) consists of random-sample consensus (RANSAC) regression.

The stacking ensemble learning framework of our study is illustrated in Figure 2, which consists of a base-level learning component (first layer) and a meta-level combining component (second layer). The prediction results of the base learner are used as features and input to the second layer. The meta-learner in the second layer uses RANSAC regression to integrate and converge the prediction results of the first-layer machine learning network. Finally, the prediction result of the second layer is used as the output result of the whole model.

### 2.4. Model Evaluation Method

**Figure 3:**
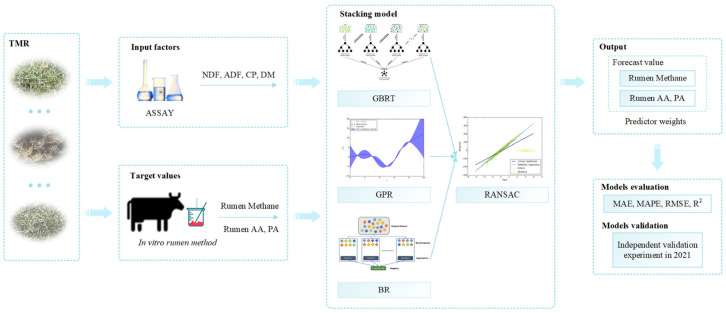
The workflow in this study was divided into four parts: inputting predictor variables and target variables; modeling through the stacking ensemble learning method; outputting results; and evaluating and validating.

The model evaluation method uses the mean absolute error (MAE), mean absolute percent error (MAPE), root mean square error (RMSE), and coefficient of determination ($R^{2}$) to measure the model evaluation performance. The formulas are as follows:

(8) $$MAE=\frac{1}{n}\overset{n}{\underset{i=1}{\sum }}\left|Y_{test}^{i}-{\hat{Y}}_{test}^{i}\right|$$

(9) $$MAPE=\frac{100}{n}\times \overset{n}{\underset{i=1}{\sum }}\left|\frac{Y_{test}^{i}-{\hat{Y}}_{test}^{i}}{Y_{test}^{i}}\right|$$

(10) $$RMSE=\sqrt{\frac{1}{n}\sum_{i=1}^{n}{\left(Y_{test}^{i}-{\hat{Y}}_{test}^{i}\right)}^{2}}$$

(11) $$R^{2}=\frac{\sum_{i=1}^{n}{\left(Y_{test}^{i}-{\hat{Y}}_{test}^{i}\right)}^{2}}{\sum_{i=1}^{n}{\left(Y_{test}^{i}-\overset{-}{Y}\right)}^{2}}$$

where n is the total number of tested samples, $Y_{test}^{i}$ is the measured value, ${\hat{Y}}_{test}^{i}$ is the predicted value, $\overset{-}{Y}$ is the average value. The independent validation experiment was conducted in 2021. And the following is a simple workflow chart (Figure 3) of this study:

## 3. Results

### 3.1. Numerical Results

The stacking ensemble learning model of this study was run on a computer with a 64-bit Windows 11 operating system, a 2.7 GHz processor, and 16 GB of RAM. The method was implemented using the Python programming language. The total time spent on the training, the testing, and the estimation stages was approximately 35 s.

To compare the prediction performance of the stacking ensemble learning model with that of a single base learner, three base learners (GBRT, GPR, and BR) were used to make predictions on the same test set. Four predictor error metrics (MAE, MAPE, RMSE, and $R^{2}$) were used to examine the prediction accuracy of different models.

From Table 2, Table 3 and Table 4, it can be seen that the $R^{2}$ values of rumen methane, AA, and PA varied in the range of 0.903 to 0.928, 0.809 to 0.888, and 0.742 to 0.924, respectively, and the stacking model gave better results compared to base learners. The prediction performance of methane is shown in Table 2. From the MAE, MAPE, RMSE, and $R^{2}$ metrics, it can be seen that the stacking model outperforms other methods. Compared with the method based on GBRT, the stacking model improves the above four metrics by 48.42%, 61.11%, 14.26%, and 2.76%, respectively. The MAPE value of the stacking model is 0.042, which is 61.11%, 51.72%, and 36.36% lower than GBRT, GPR, and BR, respectively. Table 3 and Table 4 show the prediction performances of AA and PA, respectively. In the prediction results of AA, the RMSE value based on the stacking model is reduced by 20.97%, 9.15%, and 23.42% compared to the GBRT, GPR, and BR, respectively. In the prediction results of PA, the RMSE value of the stacking model decreased by 33.45%, 45.87%, and 33.57% compared to the other three models, respectively. Combining the results given in Table 2, Table 3 and Table 4, it is clear that the stacking model shows significant improvement in all metrics compared to a single base learner.

### 3.2. Results of the Validation Experiment

The rumen fermentation parameters were predicted using the constructed models (GBRT, GPR, BR, and stacking model) to verify the feasibility and generalization ability of the rumen fermentation prediction model. The observed values and the comparison of simulated values by different models are shown in Table 5.

## 4. Discussion

### 4.1. Performance Comparison of Stacking Models and Base Learners

The purpose of this study was to predict the production of rumen fermentation products using the stacking ensemble learning method. By comparing each base learner, it is concluded that the stacking model can be used to predict the rumen fermentation parameters. In this experiment, the stacking model had better predictive performance than the base learners; it had a larger $R^{2}$ (0.928 for rumen methane, 0.888 for AA, and 0.924 for PA) and a smaller RMSE (0.968 mL/g for rumen methane, 1.975 mmol/L for AA, and 0.74 mmol/L for PA).

When performing regression prediction on the rumen fermentation parameters of dairy cows, previous studies compared multiple machine learning models and selected models through evaluating metrics, while each model has its own characteristics, strengths, and weaknesses. The stacking ensemble learning method solves the model selection problem by combining each model rather than choosing between them, while also achieving better generalization performance. In this study, GPR had a smaller $R^{2}$ and a larger RMSE when predicting PA, probably because it is difficult to estimate the aleatoric uncertainty accurately when the data are sparse. The stacking model overcomes this problem by integrating GPR with different models, improving $R^{2}$ values by 24.53%, and reducing RMSE values by 45.87%. The meta-learner minimizes the weaknesses of each base learner, and the error of GPR is likely to be compensated by other base learners, which is one reason why the stacking model works well. In future work, the stacking model can be fused with other models to further improve the accuracy of the models. Another possible reason is that the stacking model is more advantageous when dealing with small sample data. Barton and Lennox [26] use the stacking ensemble learning method to improve the prediction robustness for soft sensor development, which solved the small sample problem in the chemical industry. In livestock farming, as in the chemical industry, the small sample problem is an urgent need to be solved. The stacking ensemble learning method can improve prediction accuracy because it uses the full dataset through cross-validation, which makes it particularly suitable for small datasets where training with all the data is critical. In this study, 5-fold cross-validation was used to reduce the overfitting of the model. The third possible reason for the higher accuracy of the stacking model in this study is that the modeling data and the test data belonged to the same set of experimental data. While the independent verification experiment can further prove this. For better robustness, some extra time is consumed during the model training due to the integration of the model. However, after the model is trained, the extra time consumed in prediction is perfectly acceptable.

### 4.2. Effects of Fiber Content on Rumen Fermentation Parameters

In this study, the levels of methane, AA, and PA produced during rumen fermentation were predicted for different diets. Dietary changes induce rumen microbial community adaptations that alter fermentation patterns and contribute to changes in VFAs [27]. And changes in methane emission are generally attributed to shifts in the profile of VFAs [28]. It is extremely important to study the relationship between diet composition and rumen fermentation products (methane, AA, and PA).

Broadly speaking, rumen metabolism can be divided into three layers [29]. In the rumen metabolism of the second and third layers, VFAs are produced. The production of AA is a hydrogen-liberating reaction. Whereas the production of PA utilizes hydrogen [30]. Meanwhile, in the third layer, methanogens use hydrogen to produce methane. The higher dietary fiber content increases the production of AA and decreases the production of PA [31]. To summarize, shifting the rumen to diets with PA-dominated fermentation resulted in a decrease in methane emissions, while shifting to diets with AA-dominated fermentation resulted in an increase in methane production. And the results of the present experiment are consistent with the above conclusions.

**Figure 4:**
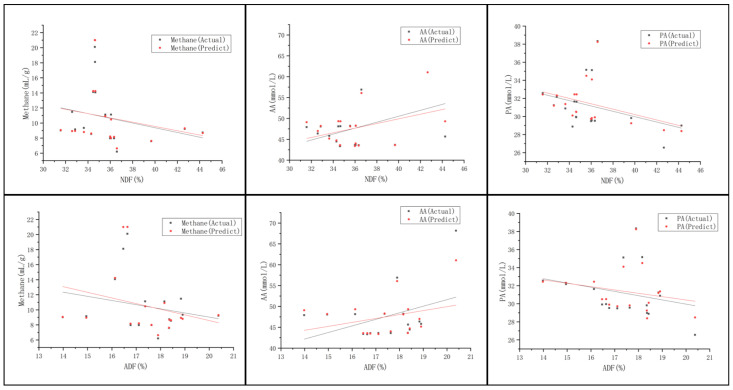
Scatter plots of actual values of methane, AA, and PA and values predicted by the stacking model at different NDF and ADF levels.

As shown in Figure 4, it can be seen that the stacking model can reflect the trend of real data to some extent. With the increase of NDF and ADF, AA showed an increasing trend and PA showed a decreasing trend, in agreement with previous studies. The results of the study by Aguerre et al. [32] showed that methane emissions per unit of dry matter intake increased with increased fiber content in the diet. However, the decrease in methane emission was not associated with expected changes in the ruminal VFAs pattern. And in the test set of this study, NDF and ADF showed no significant relationship with methane. The possible reason is that in addition to dietary fiber content, dietary fiber source and particle size are also the main factors affecting the degree of rumen fermentation and feed degradation rate. When the TMRs particle size is small, the potential NDF could have escaped rumen fermentation and entered the post-intestinal digestion [33], thus affecting rumen methane production. In addition, it may be because of the small amount of data, other influencing factors, or between-animal variation. A linear fit to the predicted values of the stacking model shows that the simulated trend of rumen production is consistent with the measured data, and our model can accurately simulate the fermentation process of TMR in the rumen of dairy cows. However, during the experiment, due to the limitation of the sampled sample TMR, the value of NDF ranges from 31.57% to 44.27%, and the value of ADF ranges from 13.98% to 20.38%. In the future, more samples will need to be collected to train and optimize the prediction model to make it useful in dairy cow production management.

### 4.3. Effects of C:F Ratio on Rumen Fermentation Parameters

The C:F ratio of the diet has a significant effect on the VFA production in the rumen and determines the type of rumen fermentation [19]. Increasing proportions of concentrate in the diet resulted in a linear increase in the concentration of PA and a linear reduction of the ratio of AA to PA [34]. In the validation experiment, the concentrate ratio of T_2_ was greater than that of T_1_, which can provide more rumen fermentation substrates and produce more PA. Therefore, the diet composition of T_2_ is more reasonable [19]. Our model also simulates the transition from rumen fermentation toward PA fermentation. As the proportion of concentrate increases, the ratio of AA to PA decreases from 3.78 to 3.05, and the proportion of PA increases from 20.88% to 24.68%.

Studies have shown that rumen methane production can be influenced by changing the C:F ratio of the diet [35]. Through in vivo and in vitro tests, high-concentrate diets reduced methane emissions from ruminants [36]. Using the stacking model to predict the methane value, the obtained T_2_-sim value is lower than the T_1_-sim value, which is consistent with the conclusions of previous studies. It is worth noting that the methane predictions of all models are slightly underestimated, and one possible reason for this analysis is that methane generation in the rumen of ruminants mainly comes from rumen microorganisms. In vitro experiments control the consistency of rumen microflora and reduce the factors affecting the model results. Another reason may be related to the different collection methods for methane. In the validation experiment, methane production was measured by the real-time in vitro fermentation system, whereas the data used in the previous modeling was collected manually and may be incomplete.

## 5. Conclusions

In this study, we propose a technique for predicting methane, AA, and PA content in the rumen of dairy cows using a stacking ensemble learning algorithm combined with in vitro fermentation technology. The prediction accuracy of the stacking model is significantly higher than that of the separate machine learning methods in all evaluation metrics, especially for small samples. The generalization ability of the model has also been proven by validation experiments. Our proposed research method provides an idea that can solve the problem of simulating rumen fermentation, which can assist the in vitro method for quantitative study of rumen fermentation products and provide guidance for optimizing diet compositions and improving feeding efficiency. 

## Figures and Tables

**Table 1:** Compositions of total mixed rations (TMRs) and conventional nutrients (DM basis).

Composition	T_1_ Ingredient, %	T_2_ Ingredient, %
Corn silage	42.76	29.31
Steam-flaked corn	18.32	29.87
Alfalfa hay	17.27	10.76
Rapeseed meal	5.94	4.56
Soybean meal	7.78	9.37
Dry corn gluten feed	2.90	6.52
DDGS	2.92	6.52
Premix	2.11	3.09
Nutrient level, %		
NEL(Mcal/kg of DM)	1.56	1.66
CP	16.39	17.62
NDF	41.24	33.61
ADF	27.52	20.03
Ash	8.12	8.05
Starch	20.26	24.86
EE	3.45	4.52
NFC	30.44	36.20

**Table 2:** Comparison of the methane prediction performances of different models.

	Models	MAE	MAPE	RMSE	*R* ^2^
methane (mL/g)	GBRT	1.018	0.108	1.129	0.903
	GPR	0.855	0.087	1.12	0.904
	BR	0.764	0.066	1.081	0.911
	Stacking model	0.525	0.042	0.968	0.928

**Table 3:** Comparison of the acetic acid (AA) prediction performances of different models.

	Models	MAE	MAPE	RMSE	*R* ^2^
AA (mmol/L)	GBRT	1.579	0.03	2.499	0.821
	GPR	1.532	0.03	2.174	0.864
	BR	1.441	0.027	2.579	0.809
	Stacking model	1.015	0.019	1.975	0.888

**Table 4:** Comparison of propionic acid (PA) prediction performances of different models.

	Models	MAE	MAPE	RMSE	*R* ^2^
PA (mmol/L)	GBRT	0.883	0.028	1.112	0.829
	GPR	1.159	0.037	1.367	0.742
	BR	0.959	0.03	1.114	0.828
	Stacking model	0.584	0.019	0.74	0.924

**Table 5:** The comparison of simulated values from different models.

	Models	T_1_-obs	T_1_-sim	T_2_-obs	T_2_-sim
methane (mL/g)	GBRT	23.11	19.44	21.94	19.43
	GPR	23.11	7.26	21.94	19.6
	BR	23.11	20.84	21.94	20.86
	Stacking model	23.11	20.8	21.94	20.44
AA (mmol/L)	GBRT	38.16	51.94	41.55	56.72
	GPR	38.16	49.23	41.55	47.83
	BR	38.16	51.9	41.55	51.07
	Stacking model	38.16	52.54	41.55	56.4
PA (mmol/L)	GBRT	10.74	14.58	14.02	17.25
	GPR	10.74	10.8	14.02	15.09
	BR	10.74	21.45	14.02	18.87
	Stacking model	10.74	13.87	14.02	18.49

T_1_ and T_2_ are TMRs with different C:F ratios, which were used in the validation experiment. T_1_-obs and T_2_-obs are the observed values of methane, AA, and PA. The simulated values (T_1_-sim and T_2_-sim) of rumen fermentation products are obtained using the previously established models.

## Data Availability

The data presented in this study are available on request from the corresponding author.
